# Comparative metabolomics analysis of different sesame (*Sesamum indicum* L.) tissues reveals a tissue-specific accumulation of metabolites

**DOI:** 10.1186/s12870-021-03132-0

**Published:** 2021-07-24

**Authors:** Senouwa Segla Koffi Dossou, Fangtao Xu, Xianghua Cui, Chen Sheng, Rong Zhou, Jun You, Koffi Tozo, Linhai Wang

**Affiliations:** 1 Oil Crops Research Institute of the Chinese Academy of Agricultural Sciences, Key Laboratory of Biology and Genetic Improvement of Oil Crops of the Ministry of Agriculture, Wuhan, 430062 China; 2 Laboratory of Plant Biotechnology and Physiology, University of Lomé, Lomé, 01 BP 1515 Togo; 3 Zhumadian Academy of Agricultural Sciences, Zhumadian, 4693000 China

**Keywords:** Sesame, Metabolome profiling, Tissue-specific metabolites, Metabolic pathway, LC–MS/MS

## Abstract

**Background:**

Sesame (*Sesamum indicum* L.) leaves, flowers, especially seeds are used in traditional medicine to prevent or cure various diseases. Its seed’s market is expanding. However, the other tissues are still underexploited due to the lack of information related to metabolites distribution and variability in the plant. Herein, the metabolite profiles of five sesame tissues (leaves, fresh seeds, white and purple flowers, and fresh carpels) have been investigated using ultra-high-performance liquid chromatography-mass spectrometry (UPLC-MS/MS)-based widely targeted metabolomics analysis platform.

**Results:**

In total, 776 metabolites belonging to diverse classes were qualitatively and quantitatively identified. The different tissues exhibited obvious differences in metabolites composition. The majority of flavonoids predominantly accumulated in flowers. Amino acids and derivatives, and lipids were identified predominantly in fresh seeds followed by flowers. Many metabolites, including quinones, coumarins, tannins, vitamins, terpenoids and some bioactive phenolic acids (acteoside, isoacteoside, verbascoside, plantamajoside, etc.) accumulated mostly in leaves. Lignans were principally detected in seeds. 238 key significantly differential metabolites were filtered out. KEGG annotation and enrichment analyses of the differential metabolites revealed that flavonoid biosynthesis, amino acids biosynthesis, and phenylpropanoid biosynthesis were the main differently regulated pathways. In addition to the tissue-specific accumulation of metabolites, we noticed a cooperative relationship between leaves, fresh carpels, and developing seeds in terms of metabolites transfer. Delphinidin-3-O-(6ʺ-O-p-coumaroyl)glucoside and most of the flavonols were up-regulated in the purple flowers indicating they might be responsible for the purple coloration.

**Conclusion:**

This study revealed that the metabolic processes in the sesame tissues are differently regulated. It offers valuable resources for investigating gene-metabolites interactions in sesame tissues and examining metabolic transports during seed development in sesame. Furthermore, our findings provide crucial knowledge that will facilitate sesame biomass valorization.

**Supplementary Information:**

The online version contains supplementary material available at 10.1186/s12870-021-03132-0.

## Background

In the latest decades, the age-old histories of East Asian countries have attracted the attentiveness of both pharmacists and academics and raised the research on the diversity and bioactivities of natural compounds. Among natural products recognized worldwide with high therapeutic values, the plant’s secondary metabolites, including flavonoids, phenolic acids, terpenoids, alkaloids, tannins, and lignans, are the most sought after [1–5]. Studies have revealed that these metabolites occur in plants in a tissue-specific manner, and their content is influenced by the genetic and environmental factors [6–9]. In sesame, the diversity of bioactive phytochemicals is still not well understood. The sesame plant tissues such as leaves, flowers, and carpels are currently underused while the global market of its seeds is increasing due to their various benefits to human health [10, 11]. The recorded pharmacological functions of sesame are mainly attributed to its specific class of lignans sesamin, sesamolin, sesaminol, sesamol, and sesamolinol [12–15]. The content of the most abundant lignan in sesame, sesamin in leaves was 1/5000 or less than that of seeds [16]. However, sesame leaves extract exhibited diverse bioactivities such as antioxidative, anti-diabetes, anti-proliferative, gastro-protective, and anti-microbial effects [17–22]. Also, Hua et al. [23] have reported the anti-tumours effect of sesame flower extract. The flowers are also used to produce perfume and cologne in Africa [10]. Therefore, we speculated a diversity of bioactive compounds and a tissue-specific metabolism in sesame.

Sesame leaves shape varied from ovate to lanceolate with pointed apices, and its flowers from purple to white [10]. Pandey et al. [24] observed a high correlation coefficient between the leaf profile and pigmentation traits. In Nigeria, sesame leaves are consumed by many rural communities as vegetables in soups [25]. In China, sesame leaves may be used in decoctions or as a poultice for medicinal purposes [10]. Given their biological activities, dried young sesame leaves are reduced into powder and commercialized in Japan as a health food supplement [17]. Nevertheless, information related to the chemical composition of sesame leaves and flowers has been limited. Kubmarawa et al. [25] reported that dry leaf of sesame is comprised approximately of 18.59 % protein, 34.04 % carbohydrate, 1.66 % oil, low tannins and phytates, and seventeen amino acids, including seven essentials. Morita [26] has detected pedaliin and pedalitin in sesame leaves, and Matsufuji et al. [20], acteoside and verbascoside. In addition, Dat et al. [18], have detected 3-epibartogenic acid, epigallocatechin, and a kaempferol derivative, and Sarma et al. [19], gallic acid, chlorogenic acid, and quercetin. The most far phytochemical study on young sesame leaves was carried by Fuji et al. [17]. Coupling HPLC and MS, they qualitatively and quantitatively identified eleven compounds, including lamalbid, sesamoside, shanzhiside methyl ester, cistanoside F, chlorogenic acid, pedalitin-6-O-laminaribioside, pedaliin, isoacteoside, pedalitin, martynoside and acteoside. According to their report, acteoside accounted for 12.9 % of dry sesame leaves and might represent the major bioactive phytochemical in sesame leaves. Regarding the sesame flowers, only six flavones, apigenin, ladanetin, ladanetin-6-O-β-D-glucoside, apigenin-7-O-glucuronic acid, pedalitin, and pedalitin-6-O-glucoside have been detected [27].

The above indicated the sesame leaves and flowers might content different classes of bioactive compounds that could be exploited for nutritional and pharmacological purposes. Thus, widely targeted metabolomic analysis involving qualitative and quantitative detection of a high number of primary and secondary metabolites participating in diverse cellular activities [28] is required for: understanding the biological abilities of individual sesame tissues, a comprehensive analysis of gene-metabolites relationships, and for elaborating a suitable valorization plan of sesame leaves and flowers. This advanced method was useful in profiling tremendous plant species such as rice, tea, and tomato [6, 28, 29]. With the huge superficies of sesame grown annually worldwide, the valorization of wasted sesame biomass would generate additional profits to small-holder farmers and many industries. Furthermore, a deep understanding of sesame plant metabolism would facilitate research on the regulation of different biosynthetic pathways and the development of high-quality therapeutics sesame products.

The present study investigated the metabolite profiles of leaves, flowers, fresh carpels, and fresh seeds of sesame using UPLC-MS/MS-based widely targeted metabolomics workflow. Our objectives were to reveal similarities and differences in the phytochemical composition among these tissues, to identify the tissue-specific bioactive metabolites, and to examine the global metabolism in the sesame plant during seed development. Our findings will provide essential information on the distribution of diverse bioactive compounds in different sesame tissues and may also help to insight into the developmental regulation of metabolism in sesame plants by integrating gene–metabolites interactions. Moreover, they could also represent practical guidance for a comprehensive use and valorization of sesame flowers and leaves.

## Results

### Metabolic profiling investigation of sesame flowers, leaves, fresh carpels, and seeds

**Fig. 1:**
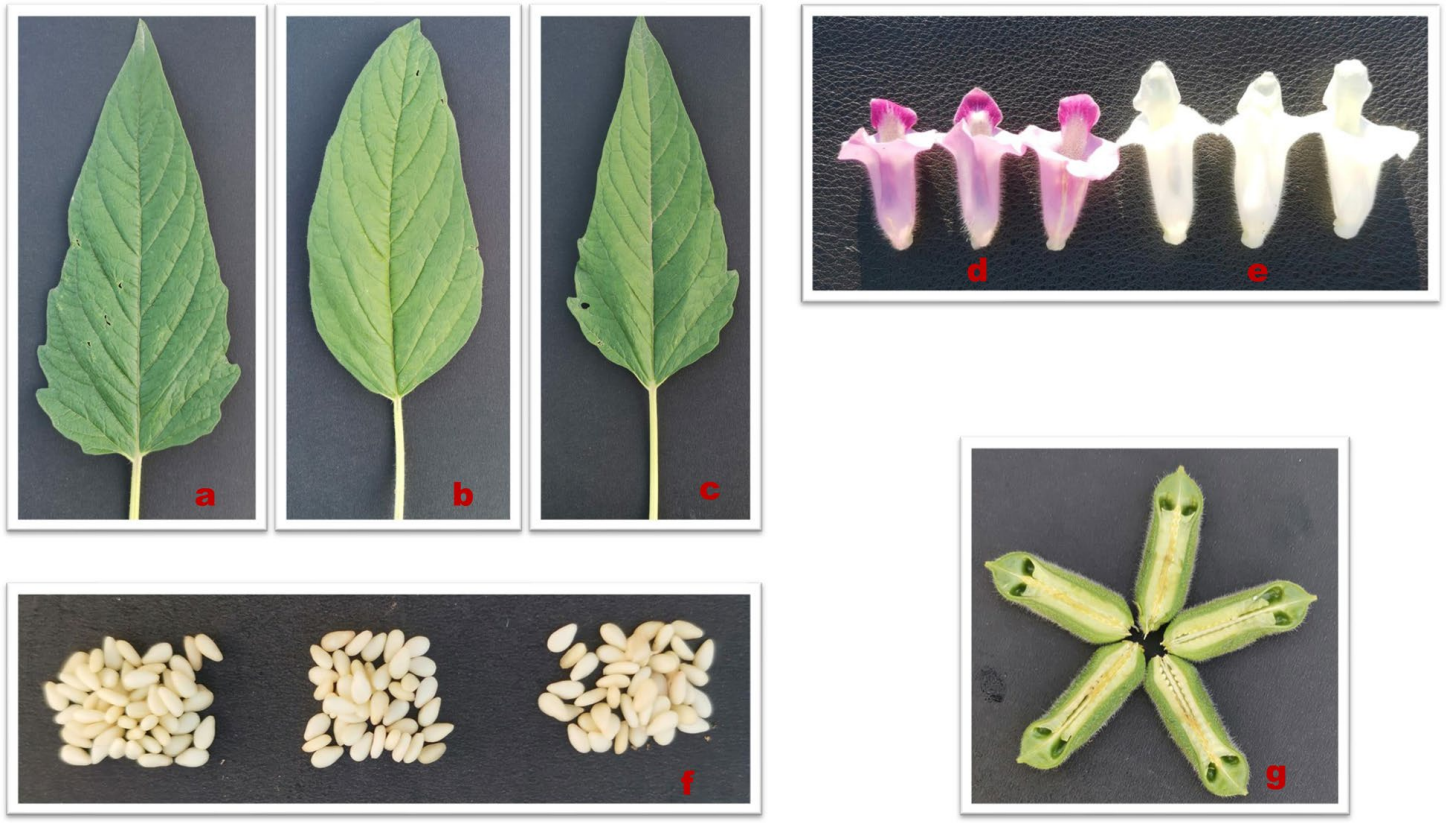
Phenotypic characteristic of five tissues of *Sesamum indicum L.* used in this study. **a**-**c** Middle leaves (ML); **d** Purple flowers (PF); **e** White flowers (WF); **f** Fresh seeds (FS); **g** Fresh carpels (FC)

Sesame flowers (white, WF and purple, PF), middle leaves (ML), fresh carpels (FC), and fresh seeds (FS) (Fig. 1) collected from Zhumadian (northern China) were subjected to ultra-high-performance LC–MS/MS-based widely targeted metabolomics analysis [28], intending to reveal the metabolites distribution and shed light on bioactive components across the different tissues. For each tissue, three materials were investigated. The samples were analyzed in both negative and positive electrospray ionization (ESI) modes in order to increase competitive ionization and accurately detect a wide range of metabolites [30]. As shown the total ion chromatograms (TICs) of the QC (quality control) samples (Fig. S1), the results are repeatable and reliable. The multiple reaction monitoring (MRM) of both positive and negative ESI modes are presented in Fig. S2. Each peak of different color represents a detected metabolite in the sample. The acquired mass spectrum peaks of the same metabolite in different samples were integrated and corrected [31]. Metabolite assignments were made based on the UPLC-MS/MS detection platform and a self-built database MWDB (Metware database). The identified metabolites were further structurally and chemically confirmed by comparing their characteristics to standard compounds whenever available.

**Fig. 2:**
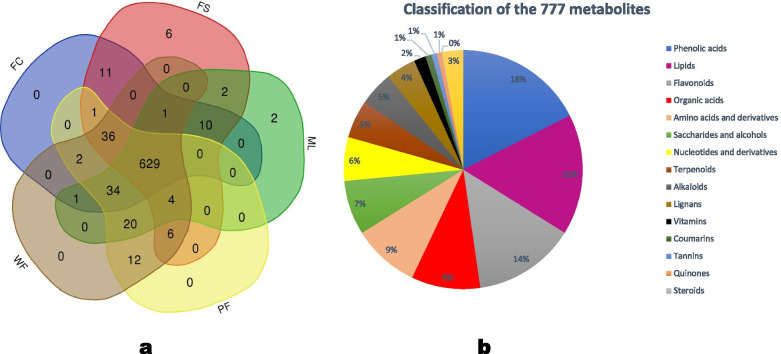
Distribution and classification of metabolites in different sesame tissues. Venn diagram result for metabolites in the different tissues (**a**), classification of 776 identified metabolites in the sesame tissues (**b**)

A total of 776 metabolites were structurally detected and annotated in the different sesame tissues (Table S1). Six hundred twenty-nine (629) metabolites were common to the five tissues (Fig. 2a). The metabolites were classified into more than 13 metabolite classes, among which phenolic acids, lipids, flavonoids, organic acids, amino acids and derivatives, saccharides and alcohol, nucleotides and derivatives, alkaloids, and lignans were the dominant (Fig. 2b). Two, six, and twelve metabolites were identified only in ML, FS, and flowers, respectively. The sesame leave’s specific metabolites comprised of a benzoquinone derivative, E-6,7-dihydroxydihydroligustilide (pmp00251), and a phthalide, senkyunolide H (pmp000253). Those of the FS included 5-Hydroxy-L-tryptophan (pme1228), 5-methyluridine (pme1187), cyclic 3ʹ,5ʹ-adenylic acid (mws0884), 15-hydroperoxyicosatetraenoic acid (mws0932), fargesin (pmn001501), and matairesinol-4,4ʹ-di-O-glucoside (Hmln001931). The specific metabolites of sesame flowers are listed in Table S2.

### Variation of metabolites in the different sesame tissues

**Fig. 3:**
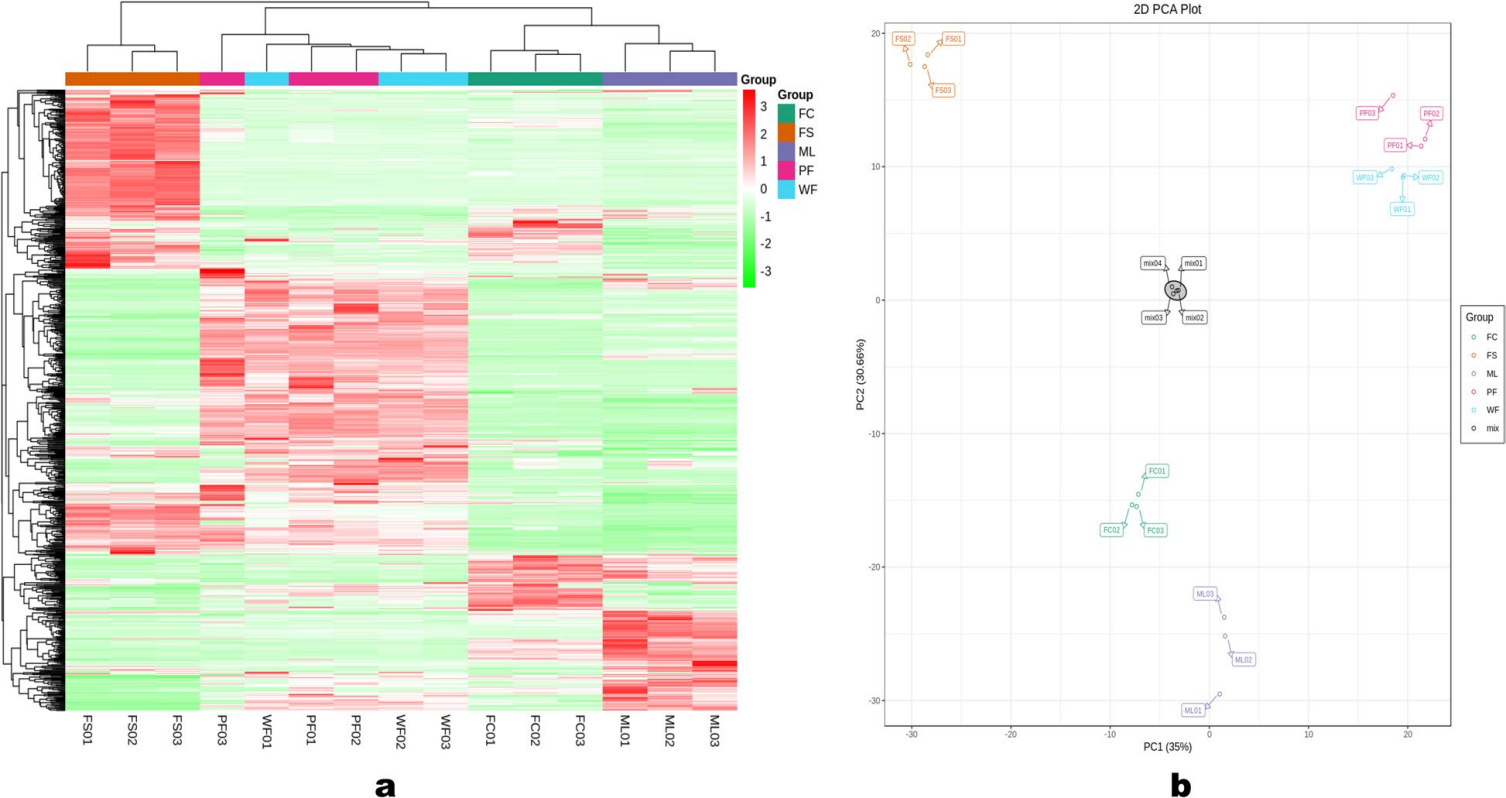
Variability of metabolites in the sesame tissues. Hierarchical cluster analysis (HCA) (**a**), and principal component analysis (PCA) (**b**)

Multivariate Data analysis was performed to examine the metabolite profiles of the different sesame tissues. In order to visualize the accumulation patterns of metabolites in the sesame tissues, we conducted the heatmap hierarchical cluster analysis (HCA) (Fig. 3a). The heatmap showed a tissue-specific accumulation of some metabolites in flowers, leaves, fresh carpels, and fresh seeds, respectively, suggesting the sesame tissues might undergo different metabolic processes. The FS were obviously separated from the other groups indicating the metabolite profile of seeds was completely different from the other tissues. The white and purple flower samples clustered together, separately from ML and FC, indicating the metabolome profiles of the white and purple sesame flowers might be similar.

Principal component analyses (PCA) of samples help to gain insight into the variability of metabolites between groups and between samples in the same group. The PCA also separated groups into four as per the HCA result along PC1 (35 %) and PC2 (30.66 %) (Fig. 3b). The QC samples were grouped together near the center of the PCA plot, confirming the repeatability and reliability of the results. The FS, ML, FC, WF, and PF samples clustered similarly to the HCA results. The phytochemical profile of the FS was completely different from those of other tissues. Furthermore, the correlation analysis also supported the observed trends of metabolites variation in the sesame tissues. As shown in Fig. S3, we observed a weak correlation between ML and FC and a strong correlation between WF and PF.

### The differential metabolites across the sesame tissues

**Fig. 4:**
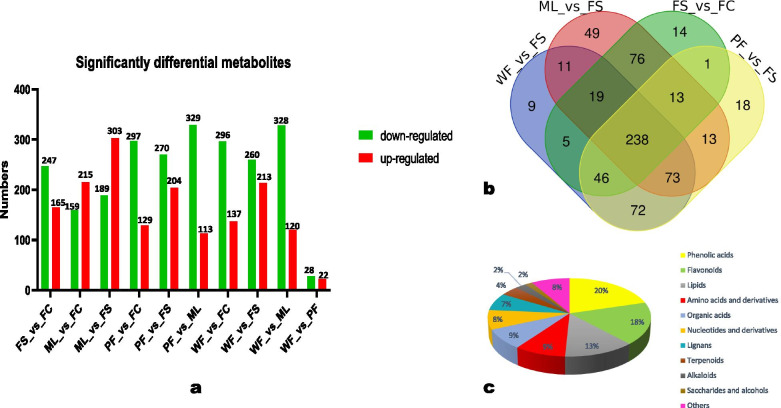
Differential metabolites across the sesame tissues. Significantly differential metabolites in the pairwise comparison between groups (**a**), Venn diagram exhibiting the number of the key metabolites that varied among the different tissues (**b**), and the classification of the 238 key metabolites (**c**)

In order to identify significantly differential metabolites between the sesame tissues, we carried Orthogonal Partial Least Squares-Discriminant Analysis (OPLS-DA). The score plots of the pairwise comparison between ML and FS, WF and FS, FS and FC, WF and PF, ML and FC, and WF and ML are shown in Fig. S4. The results indicated high predictability (Q2) and strong goodness of fit (R2X, R2Y) of the model. The Q2 values for all the pairwise comparisons were above 0.95 except for the comparison between WF and PF (Fig. S5). The criteria of *p* < 0.05 and VIP > 1 were applied to filter out the significant differential metabolites within each pairwise comparison. There were 492 metabolites significantly different between ML and FS, among which 303 and 189 were down-regulated and up-regulated, respectively, in leaves (Fig. 4a). Meanwhile, 412 (165 up-regulated), 448 (120 up-regulated), and 50 (22 up-regulated) significantly differential metabolites were identified between FS and FC, WF and ML, and WF and PF, respectively (Fig. 4a). These results support the tissue-specific accumulation of metabolic compounds in sesame. To identify key metabolites that varied in the different sesame tissues, a Venn diagram was constructed among the differential metabolites between WF and FS, ML and FS, FS and FC, and PF and FS (Fig. 4b). The result indicated there were 238 overlapping significantly differential metabolites. The 238 overlapping metabolites (Table S3) were then considered as the key metabolites that might be regulated differently in each sesame tissue. The classification of the key significantly differential metabolites is presented in Fig. 4c, with phenolic acids, flavonoids, and lipids as the top three classes.

### Distribution characteristics of metabolites in the different sesame tissues

**Fig. 5:**
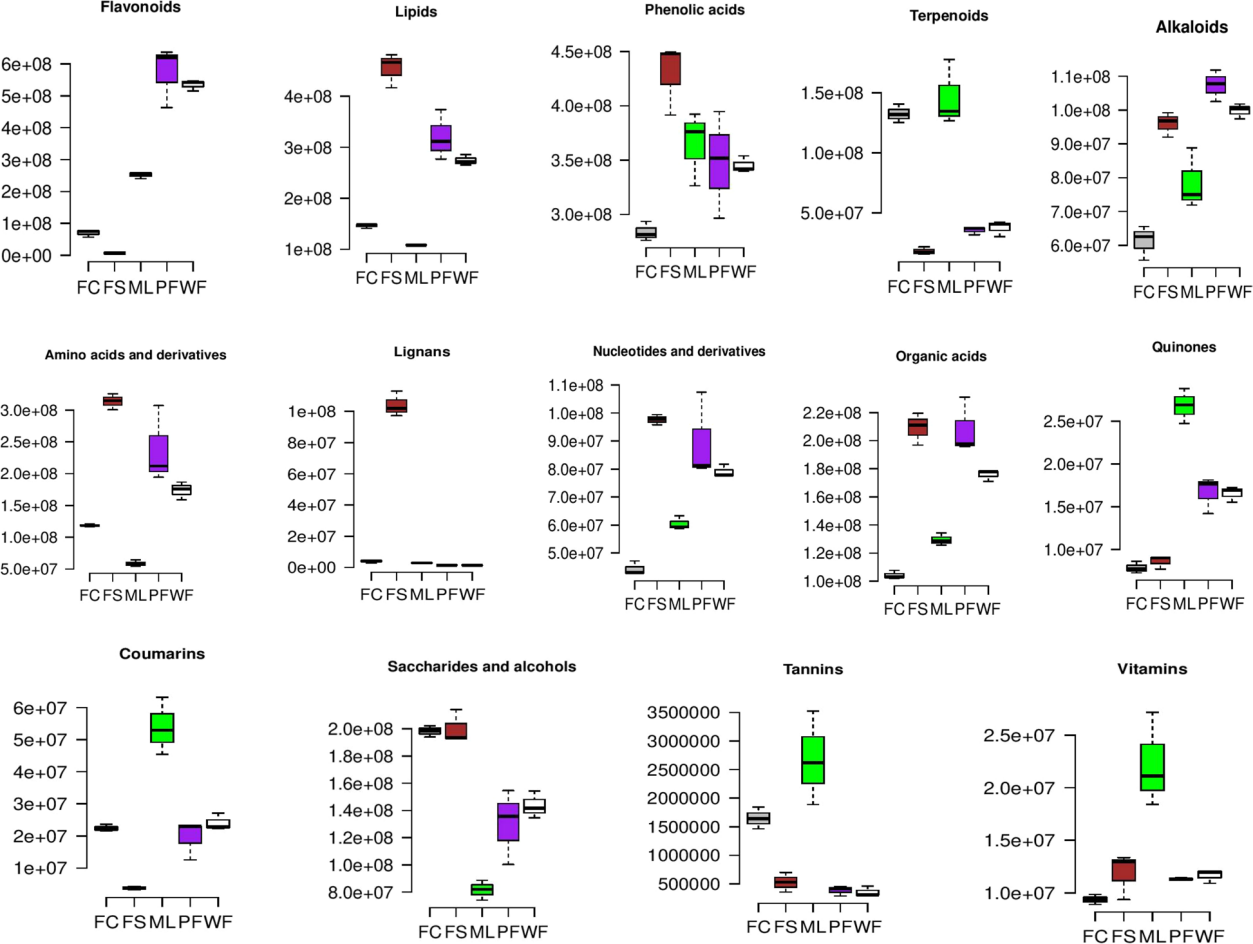
Variability of the different metabolite classes in the sesame leaves (ML), fresh carpels (FC), fresh seeds (FS), white flowers (WF), and purple flowers (PF)

**Fig. 6:**
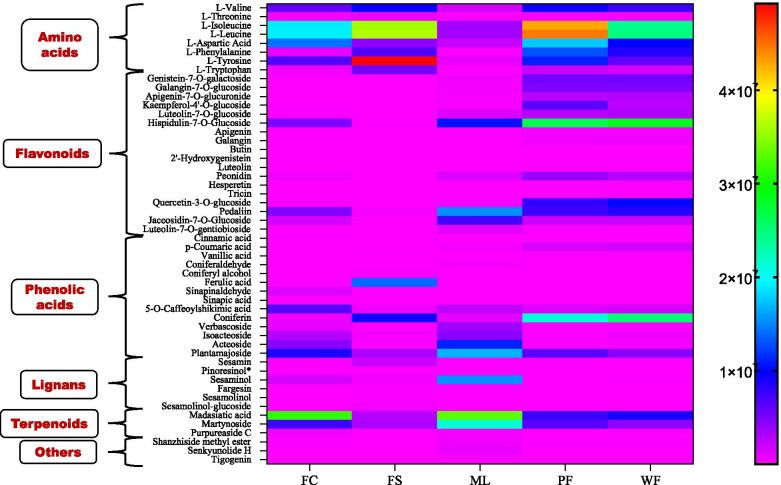
Heatmap showing the relative content of 52 selected bioactive metabolites in the different sesame tissues

To reveal the major accumulated metabolites in each of the sesame tissue, we investigated the relative content of the different categories of metabolites. As shown in Fig. 5, the relative content of flavonoids, phenolic acids, terpenoids, lipids, lignans, alkaloids, amino acids and derivatives, etc., varied in the sesame tissues. The flavonoids were highly accumulated in flowers, followed by leaves and fresh carpels. Lipids, and amino acids and derivatives exhibited a high content in FS, followed by flowers. Lignans were primarily accumulated in seeds. Leaves exhibited the highest relative content of quinones, tannins, coumarins, and vitamins. Phenolic acids were more accumulated in FS followed by ML and flowers. The FC showed a low content of both the metabolites except for terpenoids, and saccharides and alcohols (Fig. 5). For more investigation, we randomly selected 52 differential metabolites, including amino acids, flavonoids, phenolic acids, terpenoids, lignans, etc., and examined their relative content in each tissue (Fig. 6). The results indicated similar trends of metabolites accumulation in the sesame tissues. The leaves exhibited higher content of acteoside, isoacteoside, martynoside, plantamajoside, and sesaminol compared to the other tissues. Based on the report of Fuji et al. [17], stating that acteoside might be the major bioactive compound in sesame leaves, we compared its relative content with other up-regulated known bioactive metabolites in ML (Fig. S6b). The result showed the relative content of acteoside in leaves was low compared to skimmin, martynoside and sesaminol.

In order to differentiate between the white and purple flowers and determine flavonoid compounds that might be responsible for the purple coloration, we examined the content of the 50 significantly differential metabolites in the two tissues (Fig. S6a). It showed most of the differential flavonoids were up-regulated in the PF. They included five flavonols, quercetin-3-O-robinobioside (pmn001583), quercetin-3-O-glucoside-7-O-rhamnoside (Lmsp004166), kaempferol-3-O-neohesperidoside (Lmjp002867), kaempferol-3-O-glucoside-7-O-rhamnoside (Lmsp004670), and kaempferol-4ʹ-O-glucoside (Xmyp005654); one anthocyanin, delphinidin-3-O-(6ʺ-O-p-coumaroyl)glucoside (Lmpp0036620); one dihydroflavonol, phellamurin (pmp000531); and two flavones, luteolin-7-O-rutinoside (pmp000593) and apigenin-7-O-(2ʺ-O-apiosyl)(6ʺ-Malonyl)glucoside (Hmqp003435).

### The key KEGG pathways that shape the differential metabolites

**Fig. 7:**
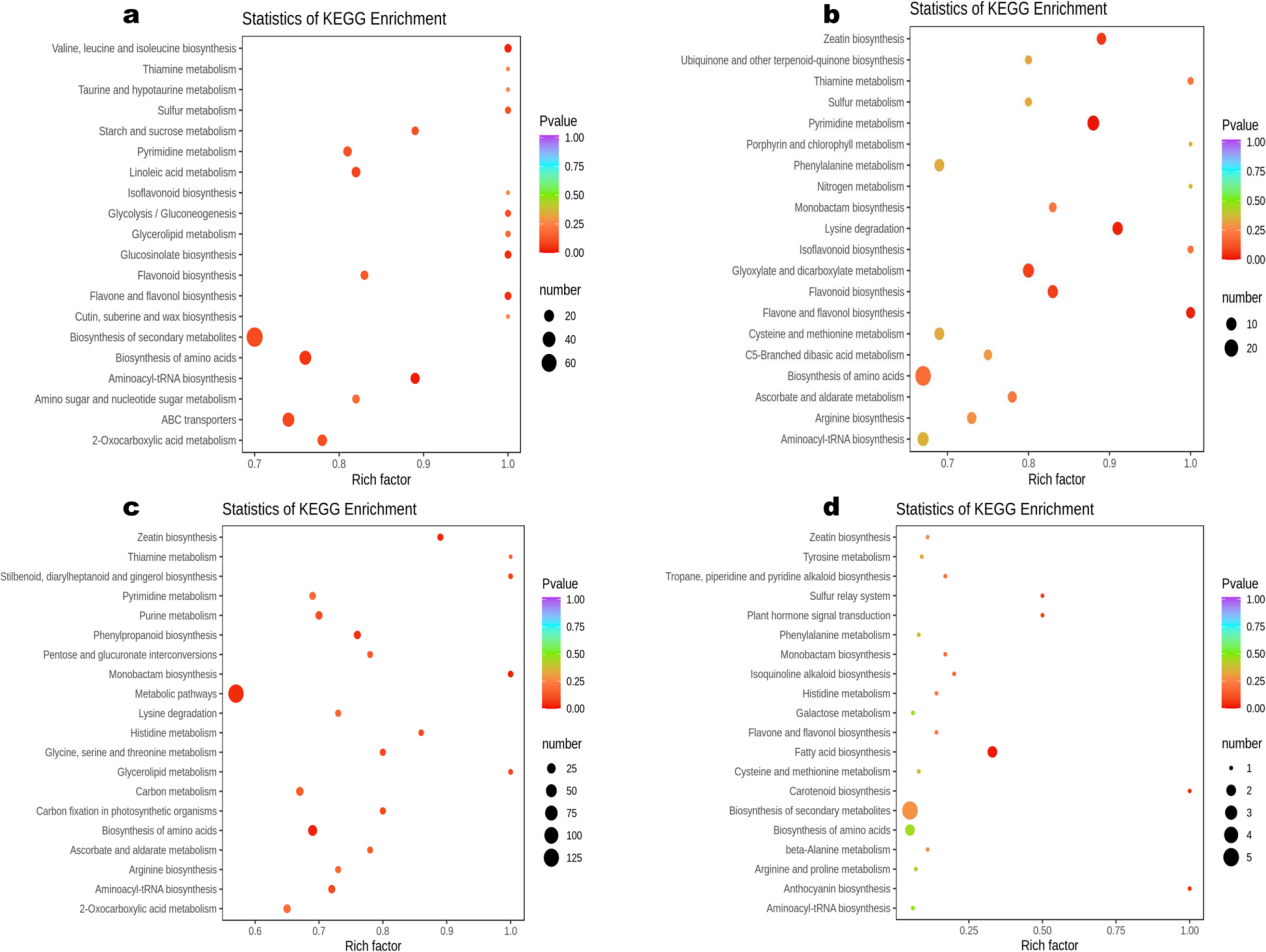
KEGG annotations and enrichment of differentially expressed metabolites of the pairwise comparison between: **a** ML vs FS; **b** WF vs FS; **c** FS vs FC; and **d** WF vs PF

**Fig. 8:**
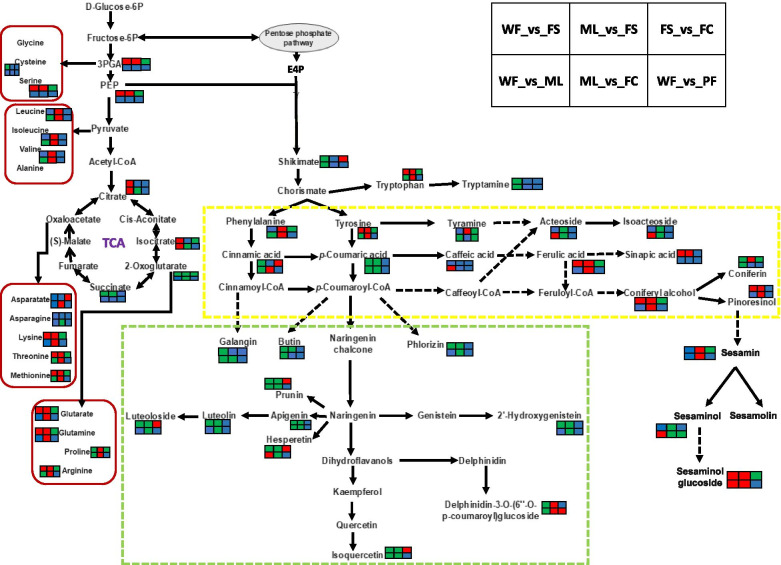
Changes in some key metabolites mapped to metabolic pathways in different sesame tissues samples pairwise comparisons. Note: The red color small rectangle indicates that the metabolite content is significantly up-regulated; the small green rectangle indicates that the metabolite content is significantly down-regulated; the small blue rectangle indicates no significant difference in that metabolite content

The KEGG pathway analysis was performed to identify the main pathways that are differently regulated in the sesame tissues. The results revealed the significantly differential metabolites between ML and both FS and FC were mostly involved in ABC transporters, biosynthesis of amino acids, 2-oxocarboxylic acid metabolism, aminoacyl-tRNA biosynthesis, flavonoid biosynthesis, phenylpropanoid biosynthesis, and biosynthesis of secondary metabolites (Fig. 7a and Fig. S7a). The differential metabolites between WF and FS occurred mainly in biosynthesis of amino acids, pyrimidine metabolism, lysine degradation, glyoxylate and dicarboxylate metabolism, flavonoid biosynthesis, and zeatin biosynthesis (Fig. 7b). As shown in Fig. 7c, the differential metabolites between FS and FC were involved mainly in metabolic pathways, amino acids biosynthesis, and phenylpropanoid biosynthesis. Figure 7d indicated that the differential metabolites between WF and PF were involved mostly in biosynthesis of secondary metabolites and fatty acid biosynthesis. The differential metabolites between WF and ML were mainly involved in ABC transporters, biosynthesis of amino acids, 2-oxocarboxylic acid metabolism, linoleic acid metabolism, phenylalanine metabolism, phenylpropanoid biosynthesis, and valine, leucine and isoleucine biosynthesis (Fig. S7b). These results indicated that the phenylpropanoid and amino acids biosynthesis pathways were the most differently regulated in the sesame tissues. Based on these results, the most relevant overlapped differential metabolites were filtered out to facilitate the overview of metabolic regulation changes (Fig. 8).

## Discussion

Recognized worldwide as a nutritionally, therapeutically, and economically important oilseed crop, sesame plants produce in high levels various bioactive compounds, among which lignans and free fatty acids were more studied [15, 16, 32, 33]. The sesame leaves and flowers showed various pharmacological abilities, but little is known about the diversity of bioactive compounds in the plant. Thus, in this study, we investigated the distribution and variability of metabolites in five sesame tissues, including leaves, fresh carpels, fresh seeds, white and purple flowers.

Totally, we identified 776 metabolites in the different sesame tissues. HCA and PCA results indicated the metabolite profiles of the sesame leaves, flowers, fresh carpels, and fresh seeds differed significantly. Notably, they showed that the phytochemical profile of the fresh seeds was far different from the other tissues. OPLS-DA revealed that there were, in general, more than 400 significantly differential metabolites in the pairwise comparison between the sesame tissues except for the comparison between the two colored flowers. The white and purple sesame flowers exhibited similar patterns of metabolites accumulation. The relative content of flavonoids in flowers was two and five-time higher than that in leaves and other tissues, respectively. Lignans were specifically accumulated in seeds. The leaves were the richest in coumarins, tannins, quinones, and vitamins. Lipids and amino acids were detected at a high level in seeds, followed by flowers. These findings indicated that metabolic compounds accumulation in the sesame plant occurs in a tissue-specific manner. The tissue-specific accumulation of metabolites in sesame was supported by the correlation analysis results. A similar accumulation pattern of metabolites has been reported in other plant species [6–8].

Flavonoids represent the third largest group of natural products widely distributed in the plant kingdom and constitute the most diverse class of polyphenol secondary metabolites [34]. They are involved in plant-environment interactions and various auto-defense processes against pathogens, ultraviolet (UV) radiation, abiotic stresses, etc. [34, 35]. One hundred thirteen (113) flavonoids, mainly flavones, flavonols, and isoflavones, were chemically and structurally identified in sesame tissues. They were highly distributed in flowers, followed by leaves, FC, and FS. KEGG enrichment analysis of the differential metabolites revealed that flavonoids, amino acids, and phenylpropanoid biosynthesis pathways were the most activated. These results suggest that the regulation of secondary metabolites biosynthesis (flavonoids, lignans, etc.), amino acids biosynthesis and metabolism might happen in a tissue-specific manner in sesame. Methylated flavones, apigenin, genistein, galangin, quercetin, luteolin, kaempferol, tricin, and their derivatives were the primary flavonoids in sesame. Quercetin, pedalitin, pedaliin, epigallocatechin, kaempferol-3-O-glucoside, 5,3ʹ,4ʹ-trihydroxy-6-methoxyflavone, apigenin, apigenin-7-O-gluconic acid, ladanetin, ladanetin-6-O-glucoside and pedalitin-6-O-glucoside have been reported in sesame leaves and flowers [17, 18, 26, 27]. Currently, many flavonoids, including apigenin, quercetin, genistein, rutin, luteolin, etc. are used in the mixed or single form as drugs or food complements to cure or prevent various diseases such as cancer, diabetes, oxidative stress, microbial infections, inflammations, amnesia and cardio-vascular dysfunctions [36–42]. Accordingly, sesame wasted flowers could be valorized as marketable tea or powder for therapeutic benefits. Glycosylation, hydroxylation, acylation, sulphation, and methylation of flavonoids enhanced their stability and solubility as well as their biological activities [34, 43, 44]. Glycosylated flavonoids were dominant in sesame flowers. The distribution of flavonoid glycosides represents excellent support to the tissue-specific accumulation of metabolites in sesame plants. It provides the first overview of the regulation of flavonoid biosynthesis in sesame tissues. This appears as a common metabolic characteristic in plants as a similar difference in the developmental regulation of flavonoids in different tissues was observed in Arabidopsis, tea, and strawberry tissues [6, 45, 46].

Flavonoids also play an essential role in plant reproduction through coloring flowers and seeds, attracting pollinators, and protecting reproductive organs [47, 48]. Among flavonoids, anthocyanins are the plant pigments primarily associated with fruits, flowers, and seeds of various colors. They can provide a wide range of colors depending on their structure, co-pigments, pH, and metal ions [34, 49]. Although the white and purple flowers showed similar trends of metabolites content compared to the other tissues, the analysis of the significantly differential metabolites indicated the two flowers could be distinguished through the content of 50 metabolites, mainly flavonoids, amino acids and derivatives, phenolic acids, and terpenoids. The 22 up-regulated metabolites in PF included five flavonols (quercetin-3-O-robinobioside, quercetin-3-O-glucoside-7-O-rhamnoside, kaempferol-3-O-neohesperidoside, kaempferol-3-O-glucoside-7-O-rhamnoside, and kaempferol-4ʹ-O-glucoside), one anthocyanin (delphinidin-3-O-(6ʺ-O-p-coumaroyl)glucoside) one dihydroflavonol (phellamurin), and two flavones (luteolin-7-O-rutinoside and apigenin-7-O-(2ʺ-O-apiosyl)(6ʺ-Malonyl)glucoside). These results suggest that a co-pigmentation between delphinidin-3-O-(6ʺ-O-p-coumaroyl)glucoside and flavonols (quercetin and kaempferol derivatives) might be responsible for the purple coloration of flowers in sesame. Delphinidin glucosides have been reported as the purple pigments in *Puerania lobata* and *Iris lutescens* flowers [50, 51]. The white flowers accumulated mostly uncolored flavonoids (flavones). The KEGG pathway enrichment analysis revealed that the differential metabolites between WF and PF occurred mostly in fatty acid biosynthesis and biosynthesis of secondary metabolites. Taken together with the relative content of lipids in flowers, we speculated that sesame flowers might accumulate a high amount of oil that can be processed. Further studies on sesame flower oil are needed to clarify this statement.

Phenolic acids and alkaloids were distributed in both the sesame tissues with relatively high content in FS and flowers. However, some phenolic acids such as acteoside, isoacteoside, verbascoside, and others exhibited the highest relative content in leaves. Fuji et al. [17] reported that acteoside content in dried mature leaves of sesame represented 12.9 %. Accordingly, they suggested that it might represent the major bioactive compound in the sesame leaves. Verbascoside also has been identified in sesame leaves [20]. Acteoside, isoacteoside, and verbascoside possess various pharmacological proprieties, including anti-inflammatory, anti-cancer, antioxidative, neuroprotective, anti-diabetes, anti-hypertensive, anti-microbial, and anti-tumours [52–55]. Sesamin, one of the major sesame lignan, was detected only in fresh seeds and fresh carpels with the highest relative content in FS. Hata et al. [16] have noticed the presence of a low amount of sesamin in sesame leaves. These suggest that the sesamin accumulation in sesame leaves might be influenced by the genotype and growing conditions. Sesaminol, a potent therapeutic lignan [15], and skimmin, an anti-diabetes coumarin [56], were detected both in all the tissues but in relatively high quantity in leaves followed by fresh carpels. The distribution of sesaminol suggests its possible involvement in sesame plant defense mechanisms or oxidative stress modulation. Moreover, leaves and fresh carpels exhibited the highest relative content of terpenoids, including martynoside and purpureaside C. Terpenoids play various ecological and physiological functions in plant life and human society through their applications in the food, pharmaceutical, and cosmetics industries [2]. The distribution of terpenoids indicate their direct or indirect implications in the sesame plant defenses and developing seeds protection. Furthermore, we identified two sesame leave’s specific phytochemicals (senkyunolide H and E-6,7-dihydroxydihydroligustilide) that have been associated with various pharmacological proprieties such as neuroprotective, anti-atherosclerotic, anti-cancer, anti-proliferative, antioxidative, cytoprotective, and anti-inflammatory effects [57, 58]. Taken together, these findings denote that sesame wasted leaves could be valorized for therapeutical purposes. Regarding fresh carpels, further biochemicals analysis are needed on mature dry carpels of sesame to accurately reveal their bioactive components.

By examining the transcriptome of developing sesame seeds and carpels, Wang et al. [59] reported a cooperative relationship between the two tissues during seed development through changes in various biological processes (transport, small molecule metabolic process, catabolic process, etc.). Herein, we observed similar relative content of saccharides and alcohols, precursors of various secondary metabolites in fresh carpels and seeds. Also, KEGG enrichment analysis associated the significantly differential metabolites between ML and both FC, FS, and flowers mainly with ABC transporters, biosynthesis of amino acids, 2-oxocarboxylic acid metabolism, aminoacyl-tRNA biosynthesis, flavonoid biosynthesis, and biosynthesis of secondary metabolites. These findings support the tight cooperation between developing sesame seeds and carpels. Moreover, they suggest dynamic exchanges or translocation of metabolic components in the sesame plant during seed development. Various metabolites transfer might occur from leaves into developing seeds via the carpel by plant’s metabolic compounds transporters, particularly ABC transporters. ABC transporters play critical roles in plant growth, reproduction, adaptation to environmental conditions by transporting actively diverse complex molecules against concentration gradients into specialized plant cells [60, 61].

## Conclusion

The present study applied the widely targeted LC–MS/MS-based metabolomics strategy to insight into the distribution of metabolites in sesame tissues. Multivariate analysis revealed diverse classes of metabolites, including flavonoids, phenolic acids, terpenoids, lipids, alkaloids, amino acids and derivatives, lignans, nucleotides and derivatives, and organic acids accumulated in a tissue-specific manner. The white and purple sesame flowers exhibited similar patterns in metabolites accumulation except for fifty metabolites, including flavonol glycosides and delphinidin-3-O-(6ʺ-O-p-coumaroyl) glucoside (anthocyanin). Therefore, we suggested that these flavonoids might be responsible for the purple coloration of sesame flowers. We found that the major metabolites in the two colored flowers were flavonoids, alkaloids, lipids, and amino acids and derivatives. Various bioactive compounds, including terpenoids, quinones, tannins, vitamins, and two specific metabolites, senkyunolide H and E-6,7-dihydroxydihydroligustilide exhibited the highest relative content in sesame leaves. Lignans were obviously related to seeds. The key differently regulated metabolic pathways were flavonoid biosynthesis, amino acids biosynthesis, and phenylpropanoid biosynthesis. We observed a cooperative relationship between developing seeds and carpels and between the two tissues and leaves. Overall, these results suggest that changes in metabolites accumulation in sesame plants are developmentary regulated, and the regulation might occur differently in each tissue. This first report on metabolites variation in sesame provides essential information for elaborating a working plan to characterize metabolic functions during sesame plant development and for the valorization of sesame leaves and flowers. Thus, an examination of gene-metabolites relationships is required to uncover regulatory elements controlling the tissue-specific metabolites biosynthesis and accumulation in sesame.

## Materials and methods

### Plant material

The sesame varieties used in the study were provided by the Oil Crops Research Institute of the Chinese Academy of Agricultural Sciences (OCRI-CAAS, Wuhan, China). They were cultivated under the same growth and experimental conditions at an experiment station of OCRI-CAAS located in Zhumadian (northern China) from May to October 2020. The middle leaves (ML), white flowers (WF), and fresh capsules were sampled from the same plants of three materials (DJ68, DJ77, and DJ80). Before sampling, the flowers were marked on the same day. When the marked capsules reached 20 days post-anthesis (DPA), all the tissues were sampled and deal with liquid nitrogen for metabolites profiling analysis (Fig. 1). The three purple flowers (PF) were similarly prepared from other three materials (28QH03, Z7, and H16). The seeds were instantly separated from capsules on ice to constituted the samples of fresh seeds (FS) and fresh carpels (FC). All the samples were conserved under appropriate storage conditions before the metabolomic profiling analysis.

### Chemicals

LC–MS gradient grade solvents methanol, acetonitrile, and acetic acid were purchased from Merck Company, Germany (www.merckchemicals.com). All other chemicals and standards were purchased from Sigma-Aldrich (St. Louis, MO, USA, www.sigmaaldrich.com/united-states.html).

### Samples preparation and extraction

The metabolic analysis was conducted following the methods described by Chen et al. [28], with some little modifications. All the sesame tissue samples were vacuum freeze-dried (Scientz-100F) and then crushed using a mixer mill (MM 400, Retsch) with a zirconia bead for 1.5 min at 30 Hz. Next, 100 mg of lyophilized powder of each sample was dissolved in 1.2 ml of 70 % methanol and vortexed vigorously for 30 s every 30 min (6 times in total). The samples were then stored overnight in a refrigerator at 4 °C to precipitate proteins. The next day, they were removed from the refrigerator and centrifugated at 12000 rpm for 10 min. The supernatants were collected separately and were filtrated using a 0.22 μm micropore membrane (SCAA-104, ANPEL, Shanghai, China). Each sample extract was conserved at 4 °C up to the UPLC-MS/MS analysis. The quality control (QC) samples were prepared by mixing each tissue sample extract.

### High-resolution UPLC analysis conditions

The sample extracts were analyzed using a UPLC-ESI–MS/MS system (UPLC, SHIMADZU Nexera X2, www.shimadzu.com.cn/; MS, Applied Biosystems 4500 Q TRAP, www.appliedbiosystems.com.cn/). The chromatographic conditions were as follows, UPLC: column, Agilent SB-C18 (1.8 µm, 2.1 mm*100 mm); The mobile phase consisted of solvent A, pure water with 0.1 % formic acid, and solvent B, acetonitrile with 0.1 % formic acid. The gradient elution system: 0.00 min, 95 % A, 5 % B; within 9 min, a linear gradient of 5 to 95 % B; 9.00 – 10.00 min, 5 % A, 95 % B; 10.00 – 11.10 min, decreased of B phase to 5.0 %; 11.10 – 14.00 min, 95 % A, 5.0 % B. The flow velocity was set at 0.35 ml per minute; the column temperature 40 °C; injection volume 4 μl. The effluent was alternatively connected to an ESI-triple quadrupole-linear ion trap (QTRAP)-MS. The m/z range was 50—1250 Da.

### ESI-Q TRAP-MS/MS

LIT and triple quadrupole (QQQ) scans were acquired on a triple quadrupole-linear ion trap mass spectrometer (Q TRAP), AB4500 QTRAP® System (AB Sciex™, Framingham, MA 01,701, USA). The system was equipped with an ESI Turbo Ion-Spray interface that operated in positive and negative ion mode and was controlled by Analyst 1.6.3 software (AB Sciex™, Framingham, MA 01,701, USA). The ESI source operation parameters were fixed as follows: ions source, turbo spray; source temperature 550 °C; ion spray voltage (IS) 5500 V (positive ion mode)/-4500 V (negative ion mode). The ion source gas I (GSI), gas II(GSII), and curtain gas (CUR) were set at 50, 60, and 25.0 psi, respectively. The collision-activated dissociation (CAD) was high. Instrument tuning and mass calibration were carried with 10 and 100 μmol/L polypropylene glycol solutions in QQQ and LIT modes, respectively. QQQ scans were acquired as MRM (multiple reaction monitoring) experiments with collision gas (nitrogen) set to medium. DP and CE for individual MRM transitions were carried with further DP and CE optimization. A specific set of MRM transitions was monitored for each period according to the metabolites eluted within the target period.

### Identification and quantification of metabolites

The metabolites were identified based on a local self-built database (MWDB, Metware Biotechnology Co., Ltd., Wuhan, China). The phytochemical was qualitatively identified based on the spectrum information, retention times relative to external standards, and mass spectra. To avoid interference, duplicate signals of K^+^, Na^+^, and NH_4_^+^ ions, isotope signals, and duplicate signals of fragment ions from other relatively large molecules were excluded. The structure of each detected metabolite was analyzed by reference to the public databases (MassBank, KNApSAcK, HMDB, MoTo DB, and METLIN). Finally, the identified metabolites were checked by comparison to the phytochemical dictionary of natural products database (CRC) and reference literature.

Metabolite quantification was conducted using the multiple reaction monitoring (MRM) mode, which consisted of triple quadrupole (QQQ) mass spectrometry analysis. In the MRM mode, the quadrupole first searched for the parent ions of target substances while screening any ions derived from substances of different molecular weights to dismiss their interference. Further, the precursor ions were fragmented to form many fragment ions. The fragment ions were then filtered through QQQ to eliminate interference from non-target ions and precisely select single-fragment ions with the desired characteristics. Next, all the obtained mass spectrum peaks were subjected to area integration. By using the MultiaQuant software (AB Sciex™, Framingham, MA 01,701, USA), we integrated and corrected the mass spectra peaks of the same metabolite in different samples. The area of each peak represents the relative content of the corresponding substance. Finally, all the integration data of the peak area were exported and saved.

### Multivariate data analysis

Before data analysis, the data quality was assessed, and substances with large deviations (CV value greater than 0.5) were eliminated. Further, Zscore was used to standardize the data. Unsupervised PCA (principal component analysis) was performed in R (version 3.5.0) using the statistics function prcomp (www.r-project.org). Prior to HCA (hierarchical cluster analysis), normalized signal intensities of metabolites (unit variance scaling) were visualized as a color spectrum. The HCA was performed with the R package pheatmap, and the results were presented as heatmaps with dendrograms. Pearson correlation coefficients (PCC) between samples were calculated using the cor function in R, and the results were presented as heatmaps.

Metabolite data were normalized by log2-transformation for Orthogonal Partial Least Squares-Discriminant Analysis (OPLS-DA). Significantly differential metabolites between groups were detected by VIP >  = 1 and absolute Log2FC (fold change) >  = 1. VIP values were extracted from OPLS-DA results. The score plots and permutation plots of the OPLS-DA were generated using R package MetaboAnalystR. To avoid overfitting, we performed a permutation test (200 permutations). Identified metabolites were annotated using the KEGG Compound database (http://www.kegg.jp/kegg/compound/). Further, the annotated metabolites were mapped to the KEGG Pathway database (http://www.kegg.jp/kegg/pathway.html). Pathways with significantly regulated metabolites were finally fed into MSEA (metabolite sets enrichment analysis). The significance of the enrichment was determined by the hypergeometric test’s *p*-values. In parallel, a comparison of the relative content of each class of metabolites and some selected metabolites in the different tissues was performed using GraphPad.prism (version 9.0.0121) and an online tool (http://www.shiny.chemgrid.org/boxplotr/).

## Supplementary Information

**Additional file 1: Table S1.** List of the identified metabolites in the sesame tissues. **Table S2.** List of the twelve sesame flower’s specific metabolites. **Table S3.** Key 238 significantly differential metabolites between the different sesame tissues.

**Additional file 2: Fig. S1.** Total ions current (TIC) overlapping map of QC samples mass spectrometry results. **Fig. S2.** MRM, metabolite detection multimodal graph of the QC sample. **Fig. S3.** Heatmap of the correlations analysis between samples. **Fig. S4.** The score plots of OPLS-DA pairwise comparisons of differential metabolites. **Fig. S5.** OPLS-DA verification diagram result for the pairwise comparison of differential metabolites. **Fig. S6.** Heatmap of the 50 differential metabolites between WF and PF (a), and Relative content of some up-regulated bioactive metabolites in sesame leaves (b). **Fig. S7.** KEGG annotations and enrichment of differentially expressed metabolites of the pairwise comparison between ML vs FC (a), and WF vs ML (b).

## Data Availability

All data generated or analyzed during this study are included in this published article and its supplementary information files. The datasets used and/or analyzed during the current study are available from the corresponding author on reasonable request.

## Abbreviations

ML: Middle leaves
FC: Fresh carpels
FS: Fresh seeds
WF: White flowers
PF: Purple flowers
UPLC: Ultra performance liquid chromatography
QC: Quality control
ESI: Electrospray ionization
HCA: Hierarchical clustering analysis
PCA: Principale component analysis
OPLS-DA: Orthogonal projections to latent structures discriminant analysis
VIP: The variable importance for the projection
