# Supplementary material for: Comparative metabolomics analysis of different sesame (Sesamum indicum L.) tissues reveals a tissue-specific accumulation of metabolites
Source: BMC Plant Biol. 2021 Jul 24;21:352. doi: 10.1186/s12870-021-03132-0 (PMC8305604; doi:10.1186/s12870-021-03132-0)
Supplement: Supplementary file 2 — Additional file 2: Fig. S1. Total ions current (TIC) overlapping map of QC samples mass spectrometry results. Fig. S2. MRM, metabolite detection multimodal graph of the QC sample. Fig. S3. Heatmap of the correlations analysis between samples. Fig. S4. The score plots of OPLS-DA pairwise comparisons of differential metabolites. Fig. S5. OPLS-DA verification diagram result for the pairwise comparison of differential metabolites. Fig. S6. Heatmap of the 50 differential metabolites between WF and PF (a), and Relative content of some up-regulated bioactive metabolites in sesame leaves (b). Fig. S7. KEGG annotations and enrichment of differentially expressed metabolites of the pairwise comparison between ML vs FC (a), and WF vs ML (b). [file 12870_2021_3132_MOESM2_ESM.pdf]

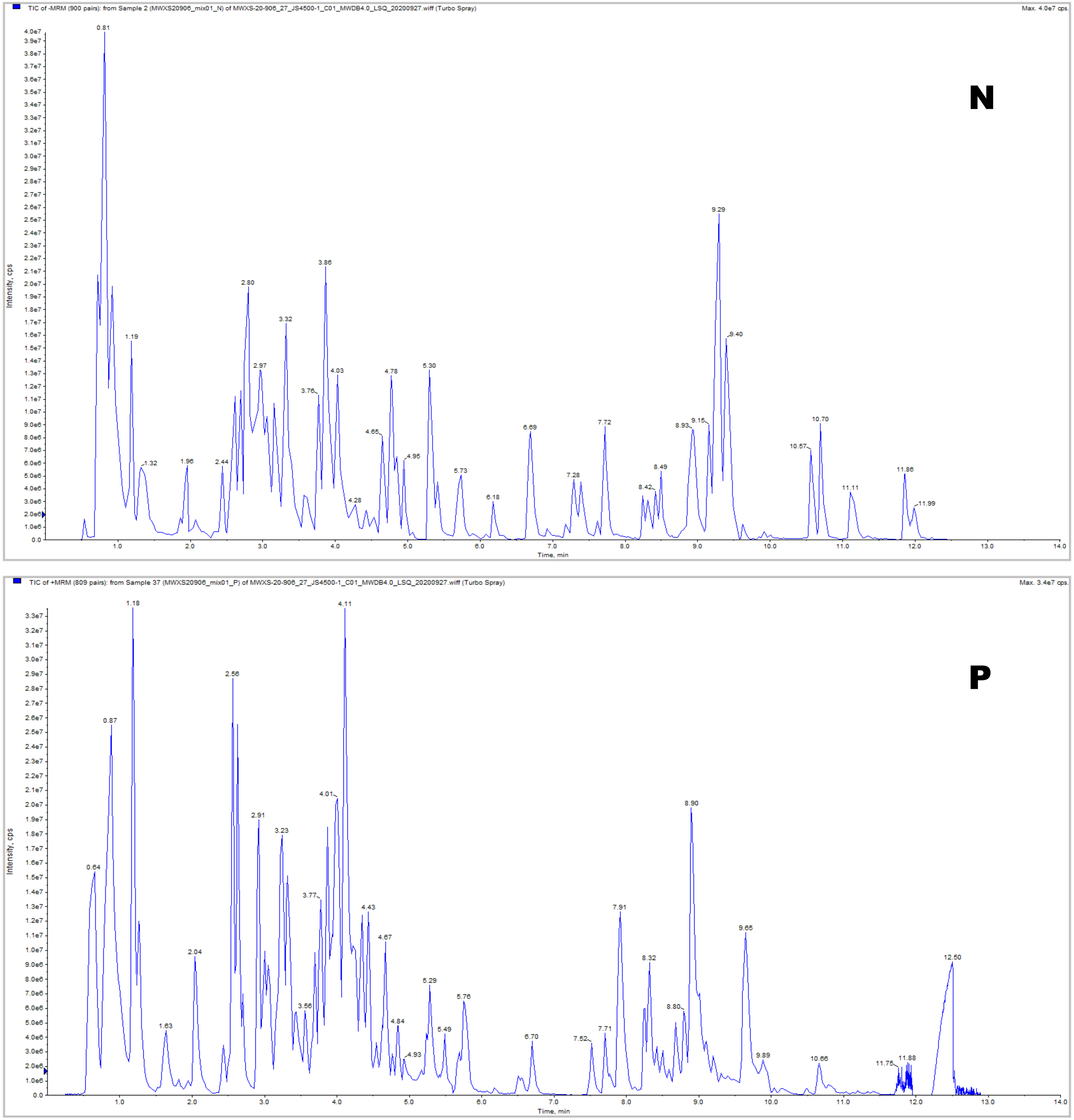

Fig. S1

Note: The abscissa is the retention time of the metabolite. The ordinate is the ion current intensity of the ion detection (the intensity units are counts per second (cps)). N stands for negative ion mode; P stands for positive ion mode.

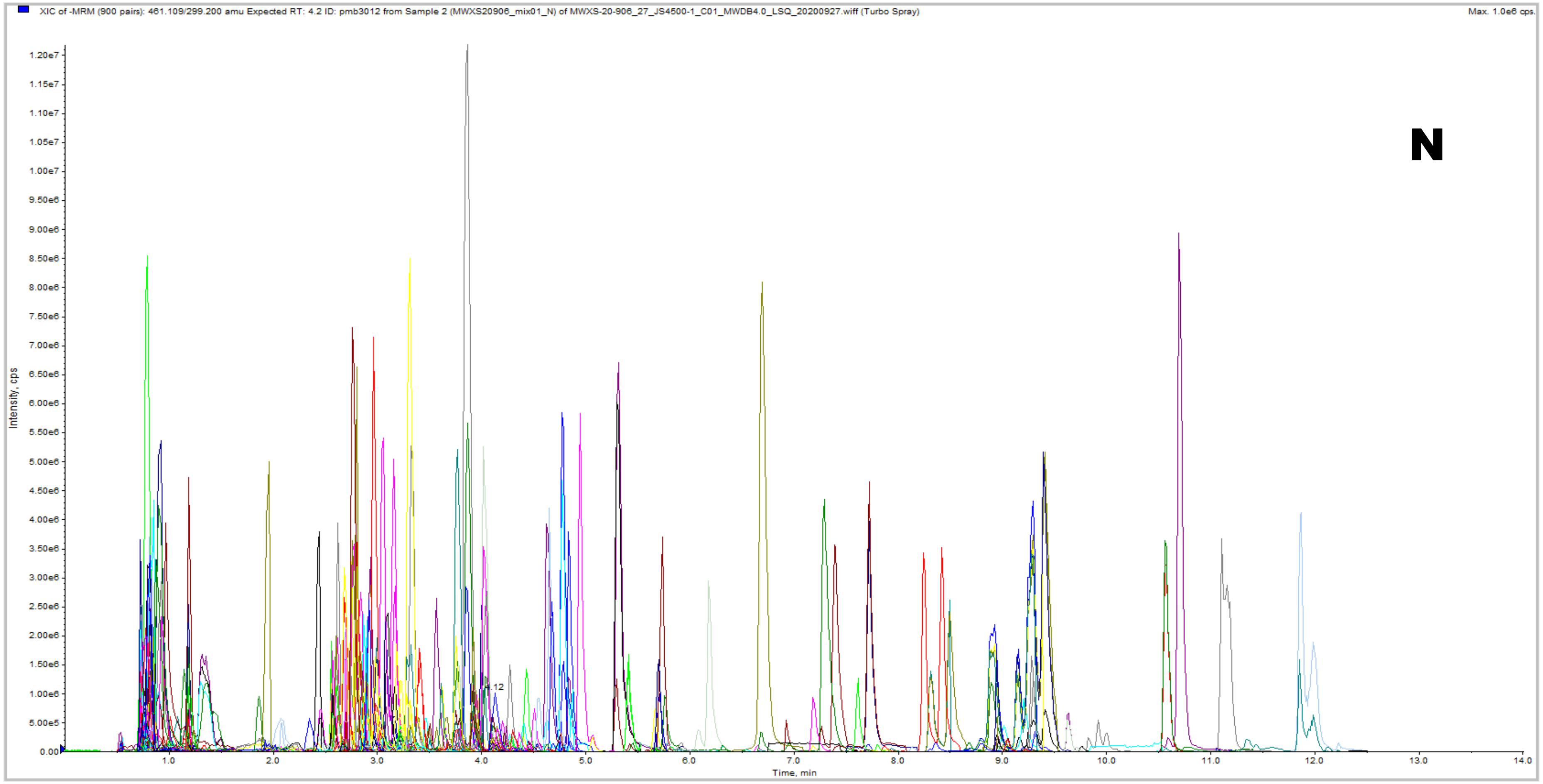

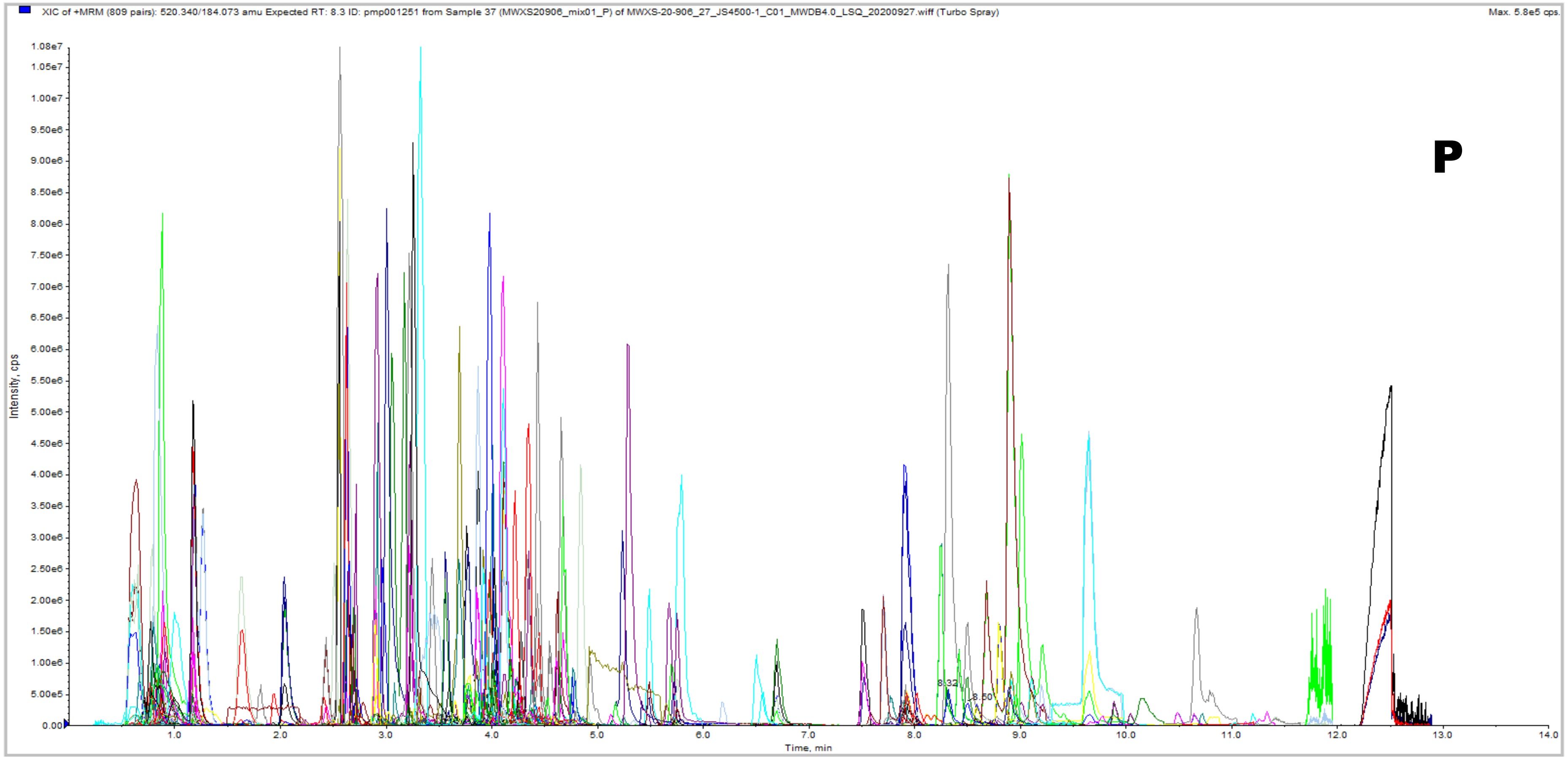

Fig. S2

Note: The abscissa is the retention time of the metabolite. The ordinate is the ion current intensity of the ion detection (the intensity units are counts per second (cps)). N stands for negative ion mode; P stands for positive ion mode.

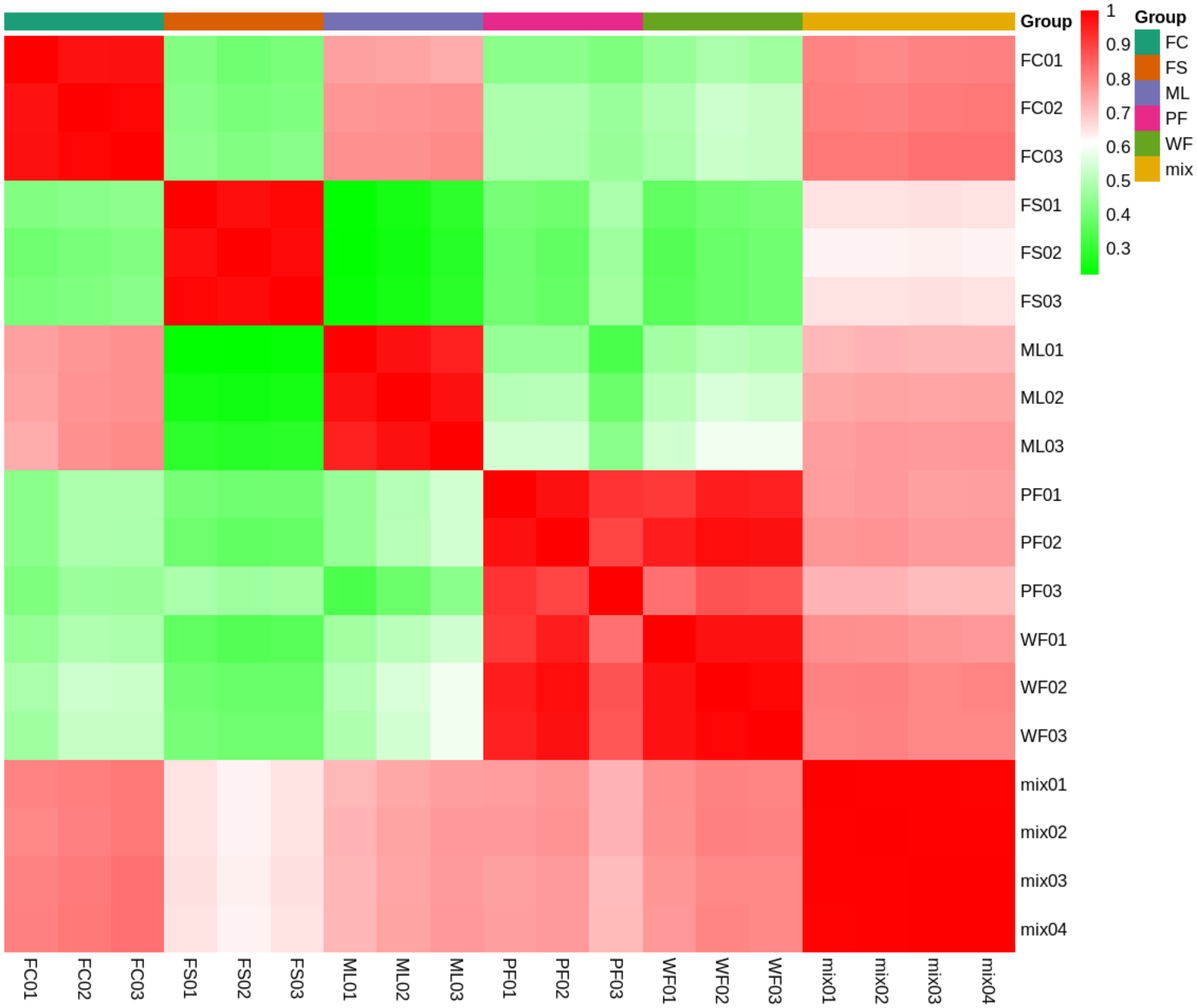

Group
FC01
FC02
FC03
FS01
FS02
FS03
ML01
ML02
ML03
PF01
PF02
PF03
WF01
WF02
WF03
mix01
mix02
mix03
mix04
1
0.9
0.8
0.7
0.6
0.5
0.4
0.3
Group
FC
FS
ML
PF
WF
mix

Fig. S3

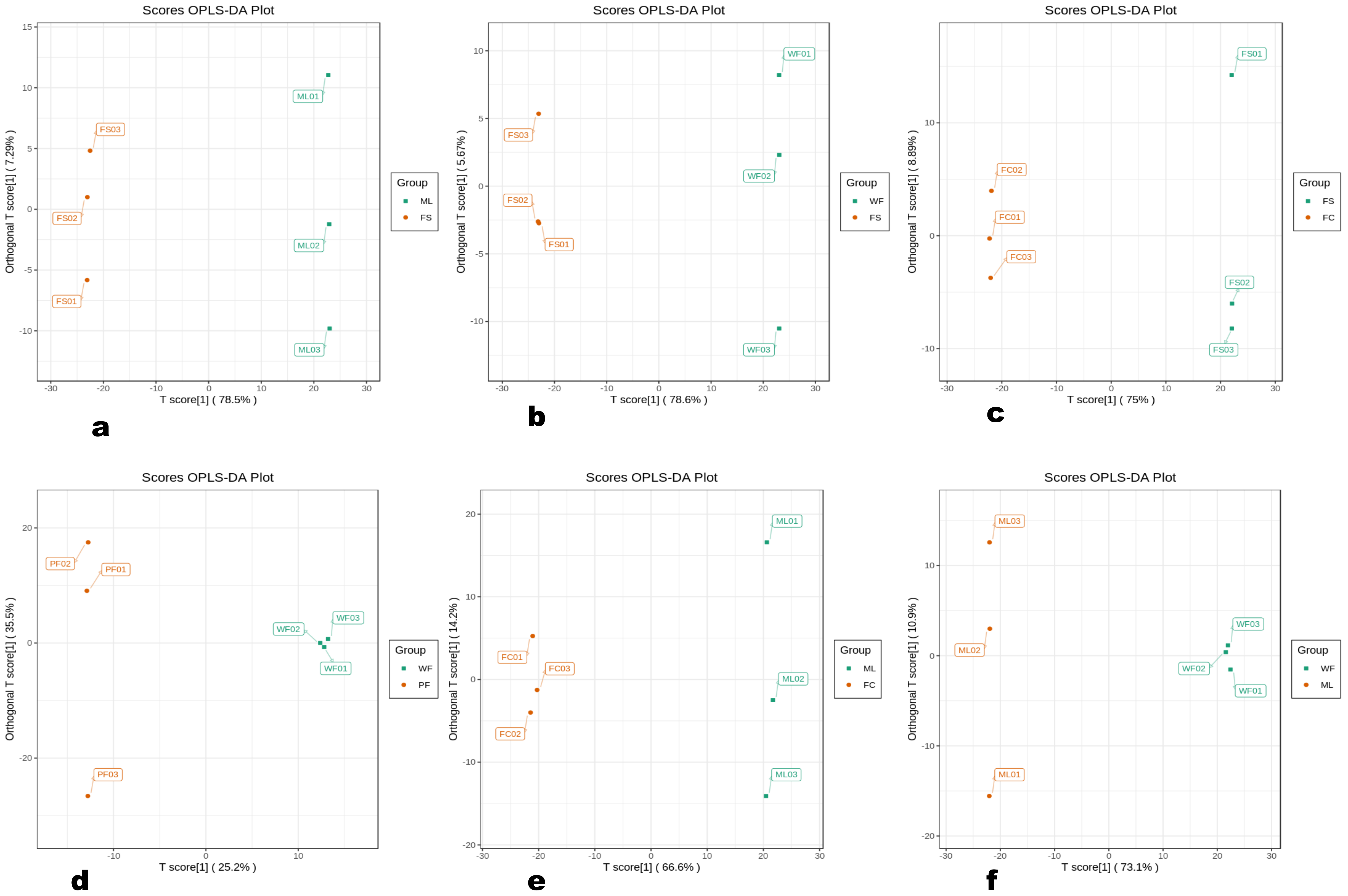

Fig. S4

Note: (a): ML vs FS; (b): WF vs FS; (c): FS vs FC; (d): WF vs PF; (e) ML vs FC, and (f) WF vs ML.

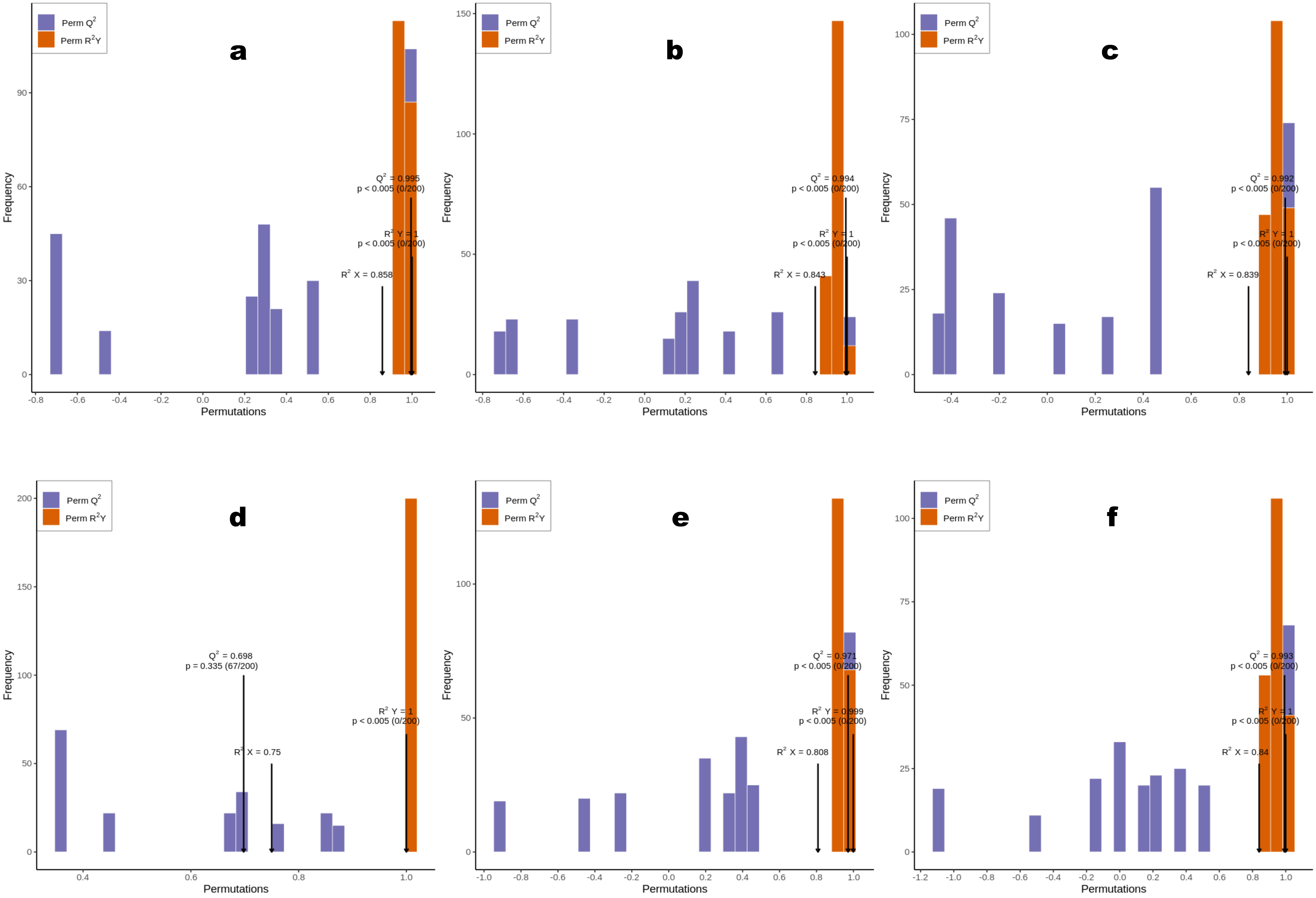

Fig. S5

Note: (a): ML vs FS; (b): WF vs FS; (c): FS vs FC; (d): WF vs PF; (e) ML vs FC, and (f) WF vs ML.

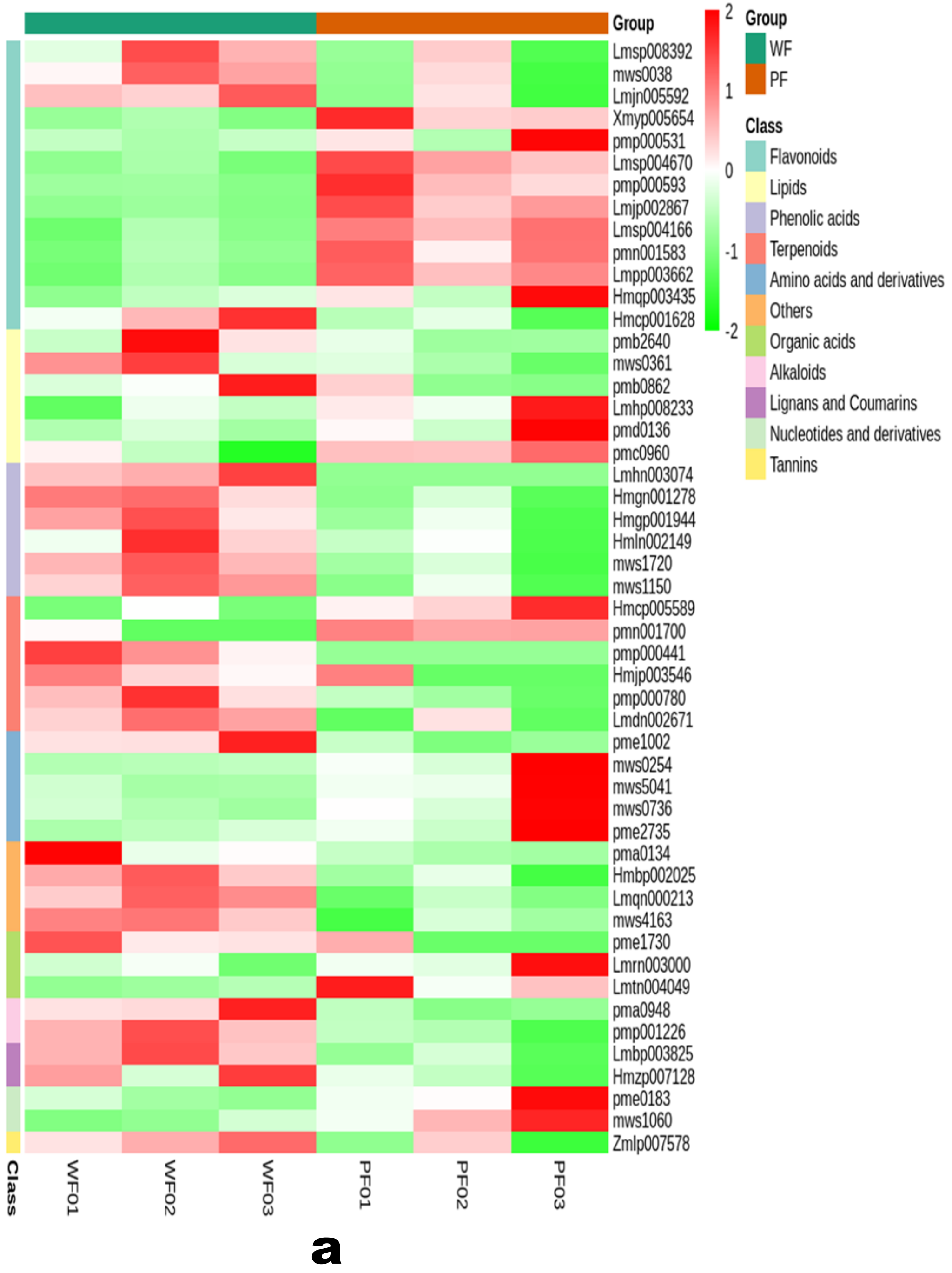
Group
2
1
0
-1
-2
Group
WF
PF
Class
Flavonoids
Lipids
Phenolic acids
Terpenoids
Amino acids and derivatives
Others
Organic acids
Alkaloids
Lignans and Coumarins
Nucleotides and derivatives
Tannins
Lmsp008392
mws0038
Lmjn005592
Xmyp005654
pmp000531
Lmsp004670
pmp000593
Lmjp002867
Lmsp004166
pmn001583
Lmpp003662
Hmqp003435
Hmcp001628
pmb2640
mws0361
pmb0862
Lmhp008233
pmd0136
pmc0960
Lmhn003074
Hmgn001278
Hmgp001944
Hmln002149
mws1720
mws1150
Hmcp005589
pmn001700
pmp000441
Hmjp003546
pmp000780
Lmdn002671
pme1002
mws0254
mws5041
mws0736
pme2735
pma0134
Hmbp002025
Lmqn000213
mws4163
pme1730
Lmrn003000
Lmtn004049
pma0948
pmp001226
Lmbp003825
Hmzp007128
pme0183
mws1060
Zmlp007578
Class
WF01
WF02
WF03
PF01
PF02
PF03
a

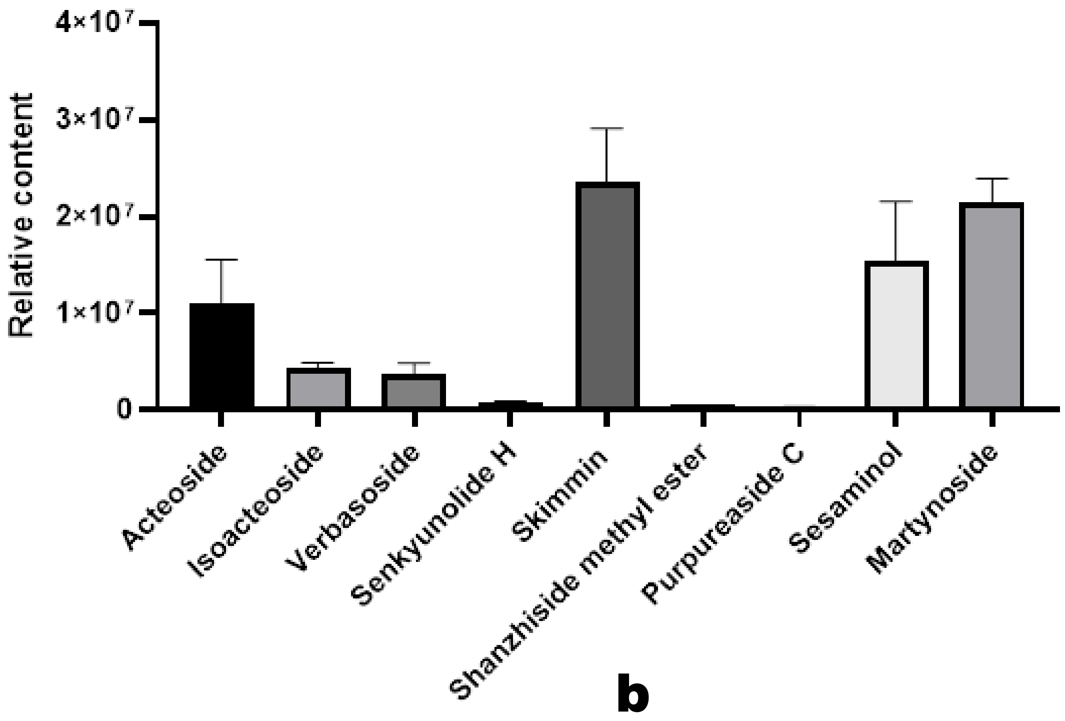
Relative content
4×10⁷
3×10⁷
2×10⁷
1×10⁷
0
Acteoside
Isoacteoside
Verbasoside
Senkyunolide H
Skimmin
Shanzhiside methyl ester
Purpureaside C
Sesaminol
Martynoside
b

Fig. S6

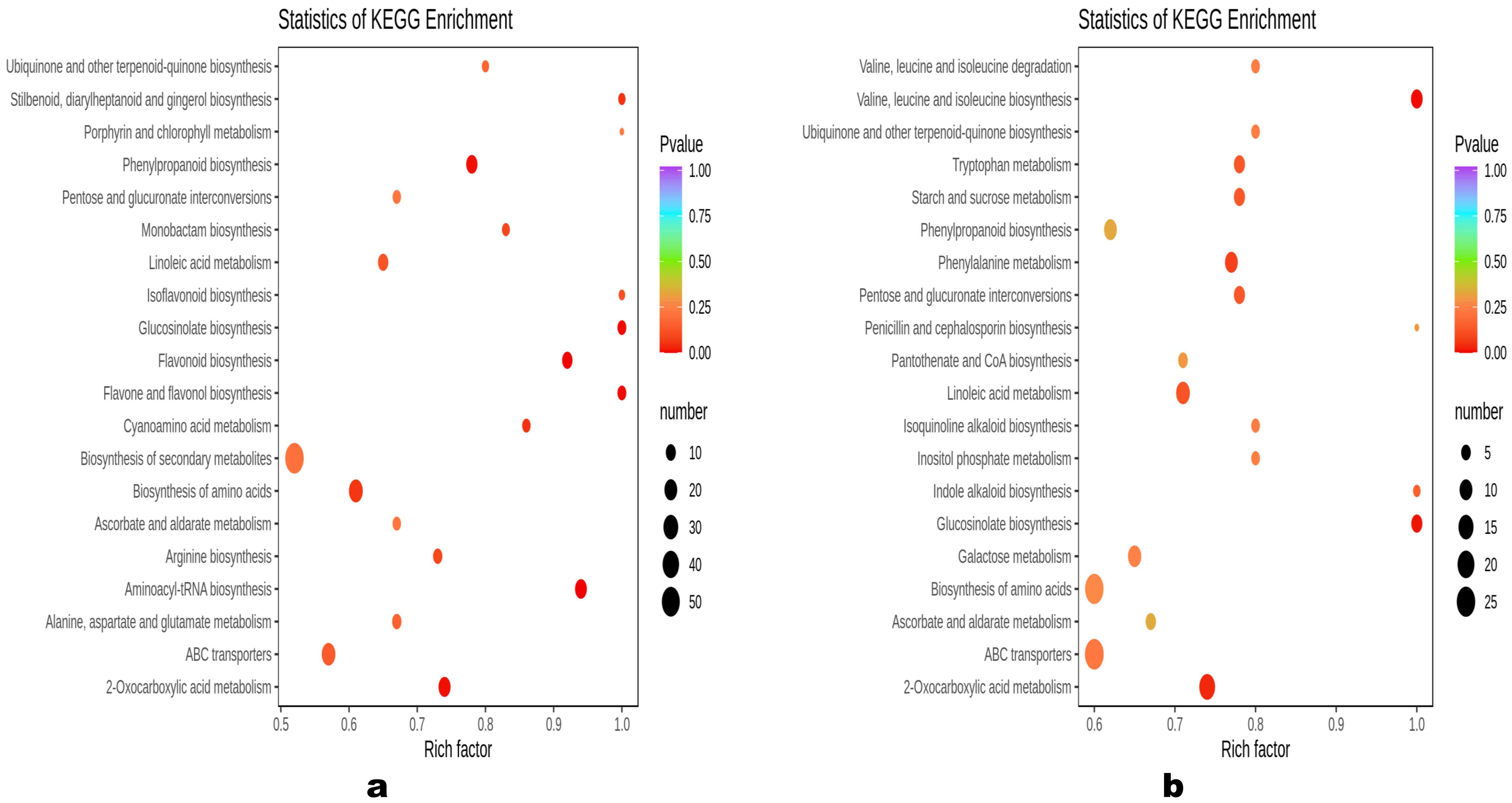

Statistics of KEGG Enrichment
Ubiquinone and other terpenoid-quinone biosynthesis
Stilbenoid, diarylheptanoid and gingerol biosynthesis
Porphyrin and chlorophyll metabolism
Phenylpropanoid biosynthesis
Pentose and glucuronate interconversions
Monobactam biosynthesis
Linoleic acid metabolism
Isoflavonoid biosynthesis
Glucosinolate biosynthesis
Flavonoid biosynthesis
Flavone and flavonol biosynthesis
Cyanoamino acid metabolism
Biosynthesis of secondary metabolites
Biosynthesis of amino acids
Ascorbate and aldarate metabolism
Arginine biosynthesis
Aminoacyl-tRNA biosynthesis
Alanine, aspartate and glutamate metabolism
ABC transporters
2-Oxocarboxylic acid metabolism
0.5 0.6 0.7 0.8 0.9 1.0
Rich factor
Pvalue
1.00 0.75 0.50 0.25 0.00
number
10 20 30 40 50
a
Statistics of KEGG Enrichment
Valine, leucine and isoleucine degradation
Valine, leucine and isoleucine biosynthesis
Ubiquinone and other terpenoid-quinone biosynthesis
Tryptophan metabolism
Starch and sucrose metabolism
Phenylpropanoid biosynthesis
Phenylalanine metabolism
Pentose and glucuronate interconversions
Penicillin and cephalosporin biosynthesis
Pantothenate and CoA biosynthesis
Linoleic acid metabolism
Isoquinoline alkaloid biosynthesis
Inositol phosphate metabolism
Indole alkaloid biosynthesis
Glucosinolate biosynthesis
Galactose metabolism
Biosynthesis of amino acids
Ascorbate and aldarate metabolism
ABC transporters
2-Oxocarboxylic acid metabolism
0.6 0.7 0.8 0.9 1.0
Rich factor
Pvalue
1.00 0.75 0.50 0.25 0.00
number
5 10 15 20 25
b

Fig. S7
